# Therapeutic Targeting of the Macrophage Immune Checkpoint CD47 in Myeloid Malignancies

**DOI:** 10.3389/fonc.2019.01380

**Published:** 2020-01-22

**Authors:** Mark P. Chao, Chris H. Takimoto, Dong Dong Feng, Kelly McKenna, Phung Gip, Jie Liu, Jens-Peter Volkmer, Irving L. Weissman, Ravindra Majeti

**Affiliations:** ^1^Forty Seven, Inc., Menlo Park, CA, United States; ^2^Institute for Stem Cell Biology and Regenerative Medicine, Stanford University, Palo Alto, CA, United States; ^3^Division of Hematology, Department of Medicine, Stanford, CA, United States

**Keywords:** CD47, AML, MDS, macrophage, immunotherapy, leukemia stem cell (LSC)

## Abstract

In recent years, immunotherapies have been clinically investigated in AML and other myeloid malignancies. While most of these are focused on stimulating the adaptive immune system (including T cell checkpoint inhibitors), several key approaches targeting the innate immune system have been identified. Macrophages are a key cell type in the innate immune response with CD47 being identified as a dominant macrophage checkpoint. CD47 is a “do not eat me” signal, overexpressed in myeloid malignancies that leads to tumor evasion of phagocytosis by macrophages. Blockade of CD47 leads to engulfment of leukemic cells and therapeutic elimination. Pre-clinical data has demonstrated robust anti-cancer activity in multiple hematologic malignancies including AML and myelodysplastic syndrome (MDS). In addition, clinical studies have been underway with CD47 targeting agents in both AML and MDS as monotherapy and in combination. This review will describe the role of CD47 in myeloid malignancies and pre-clinical data supporting CD47 targeting. In addition, initial clinical data of CD47 targeting in AML/MDS will be reviewed, and including the first-in-class anti-CD47 antibody magrolimab.

## CD47 as a Macrophage Immune Checkpoint in Myeloid Malignancies

CD47 (also known as integrin associated protein) is a transmembrane protein that mainly functions as an anti-phagocytic or “do not eat me” signal, enabling CD47-expressing cells to evade phagocytic elimination by macrophages and other phagocytes. Inhibition of phagocytosis occurs by CD47 binding to its cognate receptor Signal Regulatory Protein Alpha (SIRPα) on macrophages leading to tyrosine phosphatase activation and inhibition of myosin accumulation at the phagocytic synapse site, thereby preventing phagocytosis (1). CD47 was first identified as a tumor antigen on human ovarian cancer in the 1980s (2). Since then, CD47 has been shown to be overexpressed in multiple hematologic and solid tumor malignancies and appears to be a universal signal by which cancer cells evade the innate immune system, and specifically macrophage phagocytosis (3–8). The role of CD47 in cancer-mediated evasion of phagocytosis was first described in acute myeloid leukemia (AML) (9). In initial studies, CD47 was found to be overexpressed in both mouse and human AML compared to normal cell counterparts and its upregulation was directly tied to disease pathogenesis via macrophage evasion (3, 9). To the later point, a human CD47-negative AML cell line (MOLM13) was unable to establish disseminated disease in a xenograft mouse transplant model (9). However, forced overexpression of CD47 in MOLM13 cells led to dissemination of fulminant leukemia *in vivo* due to evasion of macrophage phagocytosis. The clinical relevance of CD47 expression was then evaluated in AML patients. Primary AML patient samples displayed increased expression of CD47 on the cell surface compared to normal cell counterparts (3). Furthermore, higher levels of CD47 mRNA expression was an independent poor prognostic factor in AML patients. Next, the therapeutic potential of CD47 blockade in AML was explored (3). Given that increased CD47 expression leads to inhibition of macrophage phagocytosis, it was hypothesized that blockade of the CD47/SIRPα interaction would lead to inhibition of the negative phagocytic signal and thus induce engulfment and elimination of leukemic cells. A blocking anti-CD47 antibody (clone B6H12) induced robust phagocytosis of primary AML patient leukemic cells *in vitro* by macrophages in contrast to IgG control or a non-blocking anti-CD47 antibody. *In vivo*, anti-CD47 antibody treatment eliminated both peripheral blood and bone marrow disease in primary patient AML-derived xenografted mice within 14 days of treatment. In addition, a humanized anti-CD47 antibody (magrolimab, previously known as 5F9) developed for clinical use demonstrated similar leukemic eradication and long term survival *in vivo* (10). While CD47 serves as a macrophage checkpoint, blockade of CD47 may also lead to an adaptive anti-tumor immune response as well. In a pre-clinical syngeneic model using ovalbumin as a model antigen, anti-CD47 antibody-mediated phagocytosis of cancer cells by macrophages led to increased priming of CD8+ T cells (11). This priming led to a memory response that protected mice from subsequent tumor challenge.

Apart from AML, similar pre-clinical findings were observed in MDS patients (8). While CD47 expression on blasts was similar in low risk MDS patient samples compared to normal cell counterparts, high risk MDS patients exhibited increased CD47 expression. It is interesting to note that increased CD47 expression from low risk to high risk MDS and ultimately AML may represent a key event of leukemic transformation from a pre-leukemic to leukemic state. Therapeutically, anti-CD47 antibody induced significant phagocytosis of MDS progenitor cells from high risk MDS patients. Thus, pre-clinical efficacy was observed with CD47 blockade in both AML and MDS patients.

## CD47 as a Leukemia Stem Cell Marker and Therapeutic Target in AML

AML is organized as a cellular hierarchy initiated and maintained by a subset of self-renewing leukemia stem cells (LSC). These LSC have been hypothesized to be a disease-initiating cell population and thus eradication of disease-initiating clones is presumably required for cure. LSC phenotype and function have been well-characterized in AML [reviewed in (12, 13)]. Clinically, LSC gene signatures have been shown to predict prognosis in AML patients, with LSC gene enrichment as an independent poor prognostic factor (14, 15). Identification and therapeutic targeting of markers of LSC is an attractive therapeutic strategy to selectively eliminate the disease-initiating cell population thus leading to potential cure. In AML patients, CD47 was identified as an LSC marker (3). CD47 cell surface protein expression was increased on CD34+CD38–CD90–Lin– leukemia stem cells (LSCs) compared to normal CD34+CD38–CD90+Lin– hematopoietic stem cell (HSC) counterparts. The specific effect of anti-CD47 antibodies on LSC elimination was explored. Anti-CD47 antibodies enabled phagocytosis of AML LSC by macrophages while sparing normal HSC *in vitro*. *In vivo*, anti-CD47 antibody therapy significantly reduced the frequency of CD34+ LSC progenitors in the bone marrow compared to control treatment. Lastly, bone marrow cells harvested from mice treated with anti-CD47 antibody failed to transplant into secondary mouse recipients thus demonstrating inhibition of LSC function *in vivo*. In summary, pre-clinical data demonstrate that CD47 is an LSC marker in AML. Thus, anti-CD47 therapies that eradicate LSCs may lead to long term remission in the clinic (16).

## Therapeutic Selectivity of CD47 Targeting Agents on Neoplastic vs. Normal Cells

**Figure 1:**
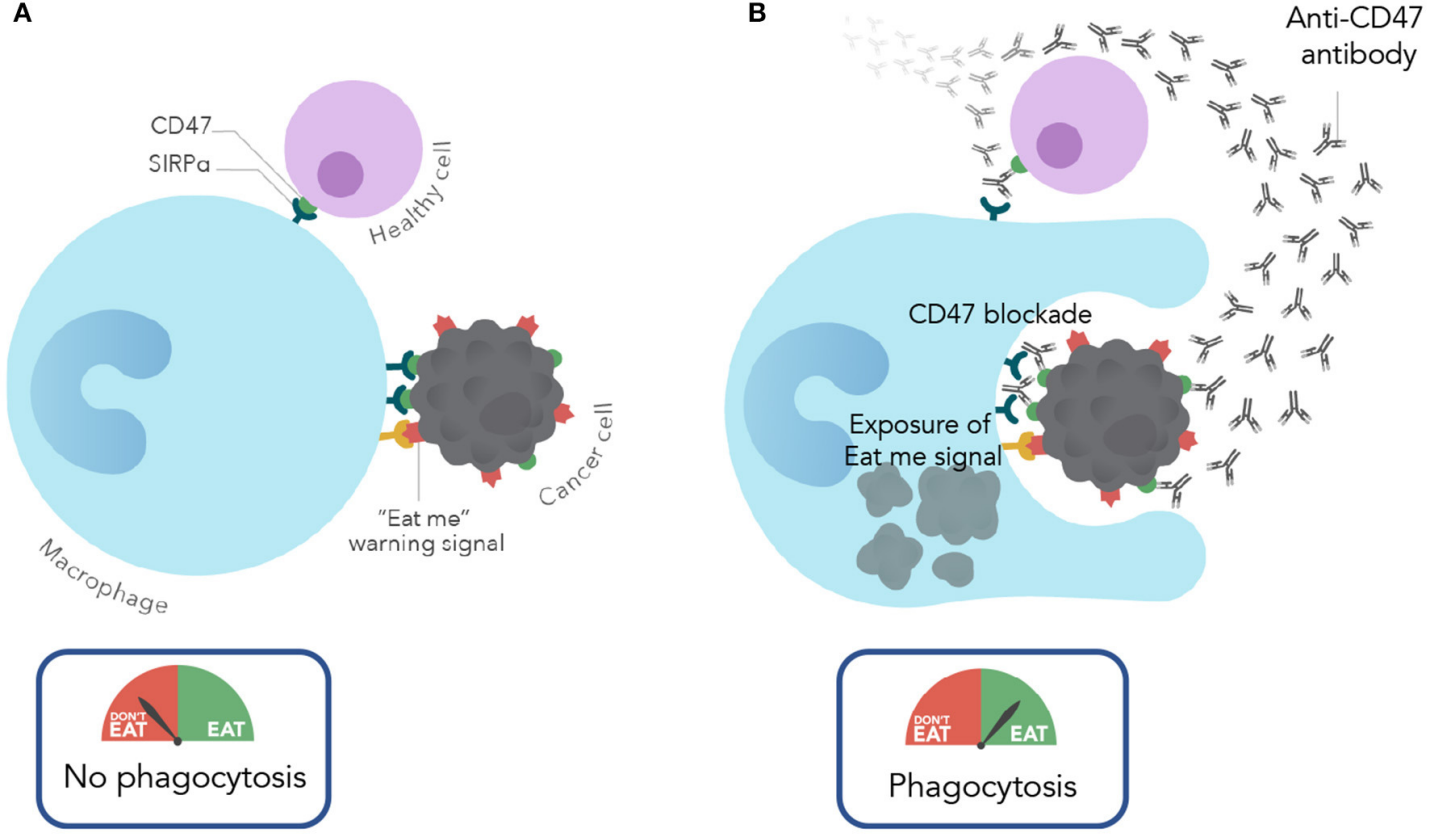
Mechanism of action of CD47 blocking antibodies. **(A)** Under normal conditions, normal and cancer cells evade macrophage phagocytosis by expressing CD47. In cancer cells CD47 is overexpressed to protect against the expression of eat me/pro-phagocytic signals. **(B)** With CD47 blockade (with an anti-CD47 antibody), cancer cells are phagocytosed due to CD47 blockade and resulting unmasking of the “eat me” signal. In contrast, normal cells are spared given the lack of expression of pro-phagocytic signals.

It is important to note that while CD47 is expressed on most cancers, it is also expressed on most normal cells. This widespread expression of CD47 on normal cells may cause concern about toxicity when considering a therapeutic approach to targeting CD47. Interestingly, in pre-clinical studies (described above), anti-CD47 antibody therapy eliminated leukemic cells, but not normal cells via phagocytosis. Thus, therapeutic targeting of cancer cells was observed with CD47 blockade. Given that CD47 is widely expressed on normal cells, what is the mechanism of this tumor selectivity? It is now known that the therapeutic targeting of CD47 depends not only on the anti-phagocytic CD47 signal, but also the expression of pro-phagocytic or “eat me” signals. During the process whereby normal cells become damaged (by stress responses, DNA damage, atypical cell division, etc.), they induce expression of pro-phagocytic signals that lead to their homeostatic removal by phagocytosis (17). This is a process termed programmed cell removal (18). It is thought that progression from these damaged normal cells to a malignant cell involves upregulation of the anti-phagocytic signal CD47 to counterbalance the expression of these pro-phagocytic signals for protection from macrophage clearance. Thus, cancer cells not only express CD47 but also pro-phagocytic signals, however, normal cells express CD47 but lack pro-phagocytic signals. Therapeutically, an anti-CD47 antibody blocks the negative phagocytic CD47 signal and unmasks and exposes an unopposed pro-phagocytic signal, thereby leading to phagocytosis (Figure 1). In contrast, blockade of CD47 on normal cells results in no phagocytosis because there is an absence of the positive “eat me” signal stimulus. While several pro-phagocytic signals likely exist, calreticulin was identified as one of the dominant “eat me” signals that functions in concert with CD47 (19).

Calreticulin is a multifunctional protein that serves several functions including calcium homeostasis, stress response, and acting as a protein chaperone, that normally resides in the endoplasmic reticulum. Upon a cell damage or stress response, calreticulin translocates to the cell surface and serves as a pro-phagocytic signal, marking cells for clearance. Calreticulin binds to its ligand low-density lipoprotein-related protein (LRP) on macrophages which leads to engulfment of the target cell. While calreticulin can be induced on cancer cells, macrophages can also secrete calreticulin that binds to cancer cells leading to macrophage phagocytosis (20). As a protective mechanism to this pro-phagocytic stimulus, cancer cells upregulate CD47 to evade phagocytosis. This mechanism is supported by three findings (19). First, cell surface calreticulin was expressed widely on multiple human cancers (AML, acute lymphoblastic leukemia, chronic myeloid leukemia, non-Hodgkin's lymphoma, bladder and ovarian cancer, and glioblastoma), but was absent on normal cell counterparts. Second, CD47 and calreticulin cell surface protein levels on cancer cells were highly correlated. Third, blockade of calreticulin led to complete abrogation of anti-CD47 antibody-mediated phagocytosis of cancer cells *in vitro*. While calreticulin is one identified pro-phagocytic signal, there are likely others including proteins involved in DNA damage and stress responses, aberrant cell surface phospholipid exposure, and aberrant cell surface protein glycosylation. In summary, pre-clinical data demonstrate that the tumor selectivity of certain anti-CD47 antibodies is based on the relative expression of the anti-phagocytic signal CD47 and pro-phagocytic signals. The scientific basis for this rationale is key for the safe and effective development of CD47 targeting therapeutics.

## Pre-clinical Rationale for Combination Strategies With CD47 Blockade in AML and MDS

**Figure 2:**
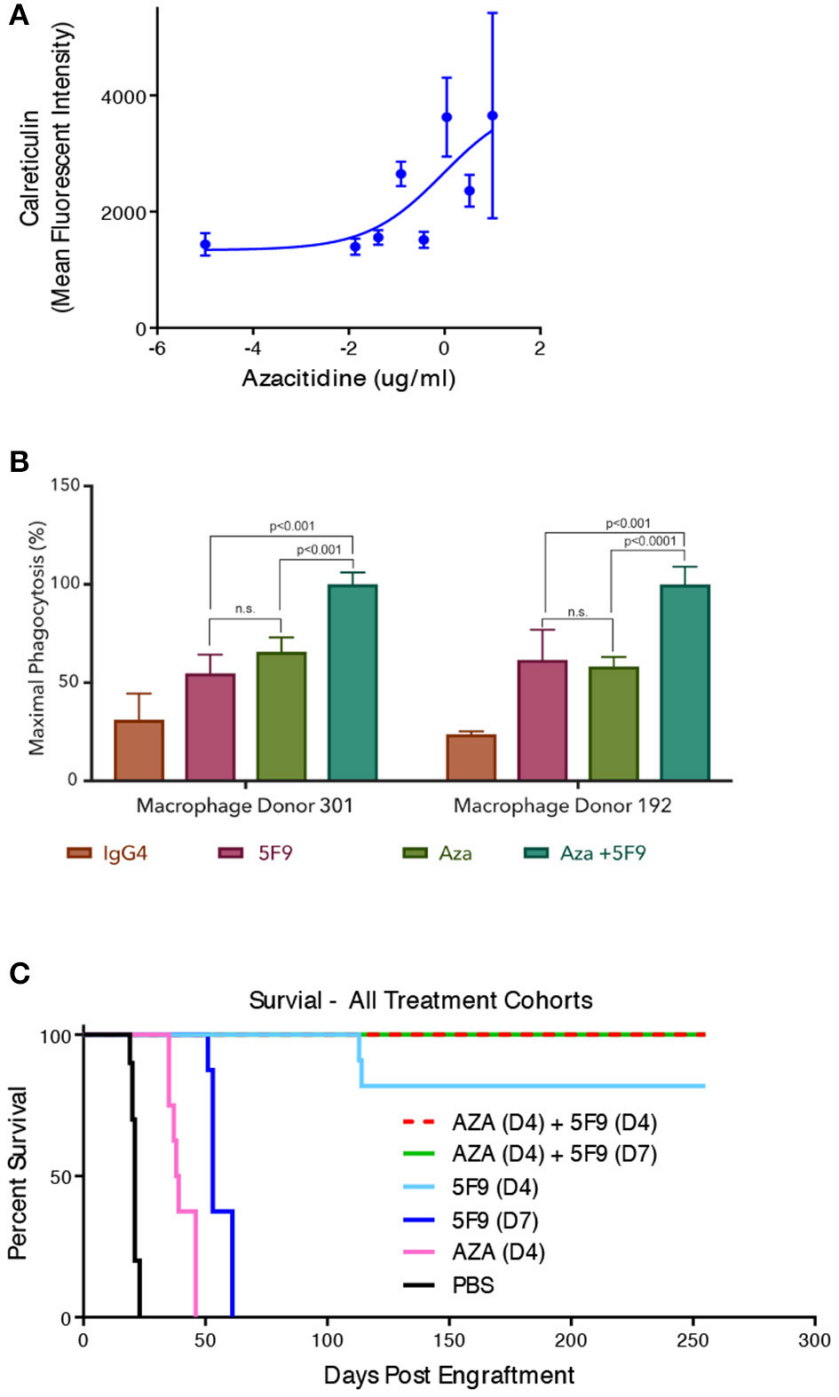
Magrolimab combination with azacitidine enhances therapeutic phagocytosis and pre-clinical efficacy in AML. **(A)** Calreticulin cell surface binding sites were assessed by flow cytometry on HL60 AML cells in the presence of increasing concentrations of azacitidine that are comparable to human exposure. **(B)** *in vitro* phagocytosis by human macrophages of HL60 cells with two different macrophage donors was evaluated in the presence of IgG4 control, 5F9/magrolimab, azacitidine (AZA), or the combination. Triplicate experiments were conducted. **(C)** HL60 mice were engrafted into immunodeficient NOD/SCID/IL2-R-gamma knockout (NSG) mice intravenously with engraftment assessed by bioluminescence imaging. Post-engraftment, mice (*n* = 10 each) were treated with PBS control, 5F9, AZA, or the combination with treatment initiated on either Day 4 (D4) or Day 7 (D7) post-engraftment as indicated. No leukemic disease was detected in mice treated with the combination that exhibited long-term survival.

While CD47 blockade has shown robust pre-clinical anti-leukemic effects as monotherapy, clinical studies to date have not shown the same degree of efficacy in patients. This observation may be due to differences in CD47 biology between mouse and human, much of which has not yet been elucidated. Thus, combination strategies with CD47 blockade are attractive routes to enhance efficacy needed for disease elimination in patients. Given that CD47 is an anti-phagocytic signal, strategies to increase pro-phagocytic signals on cancer cells can synergize with CD47 blockade. This synergy occurs through simultaneous blockade of the negative (anti-phagocytic) CD47 signal while enhancing the positive (pro-phagocytic) signal, leading to increased leukemic phagocytosis (Figure 1). It has been demonstrated that certain chemotherapies and cytotoxic agents that induce cell damage can induce pro-phagocytic signals on cancer cells (21). To investigate this strategy, a combination approach with azacitidine, a hypomethylating and cytotoxic agent, and CD47 blockade has been explored pre-clinically (22). Azacitidine induced endogenous expression of cell surface calreticulin in an AML cell line [Figure 2A; (23)]. In MDS cell lines, azacitidine was found to induce a 7 to 10-fold increase in calreticulin expression and a 4 to 6-fold increase in CD47 expression *in vitro* (24). The combination of azacitidine and the anti-CD47 antibody magrolimab lead to significantly higher macrophage-mediated phagocytosis of AML cells *in vitro* compared to either single agent alone (Figure 2B). Importantly, magrolimab combined with azacitidine led to a near 100% long term remission rate in an aggressive AML xenograft model (Figure 2C). These pre-clinical data led to the initiation of a Phase 1b clinical trial of magrolimab in combination with azacitidine in AML and MDS patients (NCT03248479). In addition to combination strategies with cytotoxic agents, other therapeutic modalities can also provide pro-phagocytic signals which is a subject of ongoing investigation.

## Clinical Experience of CD47 Targeting Agents in Cancer

Since the discovery of CD47 as a therapeutic target in AML, subsequent studies have validated the role of CD47 across multiple cancer types. Consequently, several CD47 targeting agents have entered the clinic for a number of cancer indications spanning both hematologic malignancies and solid tumors (Table 1). While magrolimab (Forty Seven, Inc.) was the first CD47 targeting agent to enter clinical development, several other CD47/SIRPα targeting agents have also recently entered the clinic, highlighting the high degree of enthusiasm for targeting this pathway. Several different approaches to targeting the CD47 pathway have been taken, including direct blocking of CD47 or its macrophage receptor SIRPα. These approaches may result in different safety and efficacy profiles, which remain to be fully understood in the clinical setting.

**Table 1:** CD47/SIRPα targeting agents in clinical development in the US.

**Compound (company)**	**Molecule**	**Clinical start date**	**Stage**	**Cancers studied**
Magrolimab/5F9 (Forty Seven, Inc.)	Anti-CD47 antibody	2014	Phase 2	MDS, AML, NHL, CRC, ovarian cancer, bladder cancer
CC-90002 (Celgene)	Anti-CD47 antibody	2015	Phase 1b	NHL, AML, MDS
CC-95251 (Celgene)	Anti-SIRPα antibody	2019	Phase 1	CRC, NHL
TTI-621and TTI-622 (Trillium Therapeutics)	Wildtype SIRPα fusion proteins	2016, 2018	Phase 1a/1b	NHL, CTCL, HL, MM
ALX148 (ALX Oncology)	High affinity SIRPα fusion protein	2017	Phase 1	Solid tumors, NHL
SRF231 (Surface Oncology)	Anti-CD47 antibody	2018	Deprioritized	Solid tumors and heme malignancies
IBI188 (Innovent)	Anti-CD47 antibody	2019	Phase 1	Solid tumors, lymphoma
AO-176 (Arch Oncology)	Anti-CD47 antibody	2019	Phase 1	Solid tumors
BI 765063/OSE-172 (Boehringer Ingelheim/OSE Immunotherapeutics)	Anti-SIRPα antibody	2019	Phase 1	Solid tumors
TG-1801/NI_1701 (TG Therapeutics/Novimmune)	CD47/CD19 bi-specific antibody	2019	Phase 1	NHL
TJC4 (I-Mab)	Anti-CD47 antibody	2019	Phase 1	Solid tumors, lymphoma

*MDS, myelodysplastic syndrome; AML, acute myeloid leukemia; NHL, Non-Hodgkin's lymphoma; CRC, colorectal cancer; CTCL, cutaneous T-cell lymphoma; HL, Hodgkin's lymphoma; MM, multiple myeloma*.

## Approaches to Optimizing the Therapeutic Window of CD47 Targeting Agents to Minimize Toxicities

CD47 is ubiquitously expressed on normal cells, which can present a major concern for potential toxicity with CD47 targeting agents. As mentioned previously, selective elimination of cancer cells is dependent on the presence of pro-phagocytic signals, like calreticulin, on cancer cells that are absent on normal cells. Among normal cells, red blood cells (RBCs) are a notable exception, in that they express pro-phagocytic signals in certain settings and can be susceptible to elimination by CD47 blockade. CD47 was previously identified as a major homeostatic regulator of RBC turnover and a marker of self and protective mechanism against RBC clearance (25). As RBCs age and near their 120 day lifespan, they lose CD47 and gain the expression of pro-phagocytic signals, which direct their homeostatic cell clearance by the splenic reticuloendothelial system. Thus, CD47 blockade has the potential to accelerate RBC clearance by unmasking of pro-phagocytic signals, leading to anemia when administered to patients. For CD47 targeting agents in clinical development, various strategies have been employed to mitigate this on target toxicity. Approaches have been employed mainly for two particular agents: magrolimab (Forty Seven, Inc.) and TTI-621 (Trillium Therapeutics). Notably, magrolimab has successfully used a priming and maintenance dose strategy to mitigate anemia. Because aged RBCs are particularly susceptible to clearance by CD47 blockade, an initial low (priming) dose of 1 mg/kg was administered to eliminate aged RBCs selectively while sparing younger red cells, which lack pro-phagocytic signals. This priming dose leads to a predictable and transient mild anemia, followed by a compensatory reticulocytosis (generation of new RBCs) that shifts the overall age of RBCs to a younger population. These young RBCs do not express significant pro-phagocytic signals, and are thus unaffected by magrolimab. Furthermore, pre-clinical data demonstrate that RBCs exposed to the initial priming dose rapidly shed CD47 protein from the cell surface becoming CD47 negative through a process called RBC pruning (26). Thus, these pruned RBCs are unaffected by subsequent magrolimab dosing To this point, subsequent higher maintenance doses at therapeutic ranges can be administered as soon as 1 week after the priming dose without worsening or recurrence of anemia in patients with multiple cancer types (10, 27, 28). This RBC antigen loss or pruning is similar to what has been observed with daratumumab, an anti-CD38 monoclonal antibody which is approved for multiple myeloma (29). However, the exact molecular mechanism for CD47 antigen loss on RBCs by magrolimab is still being elucidated (26). In addition to a priming/maintenance dose strategy, another approach to mitigate RBC toxicity has been to reduce affinity of CD47 targeting agents to RBCs. This strategy is exemplified by TTI-621, which is a SIRPα-Fc fusion protein engineered for minimal binding to human RBCs, resulting in minimal anemia. However, significant dose-limiting thrombocytopenia has been observed with administration of TTI-621 to patients that may be related to use of an activating IgG1 isotype (30). Lastly, antibodies that target the macrophage ligand SIRPα may also lead to minimal anemia as SIRPα expression is generally restricted to immune effector cells with absent expression on RBCs. In summary, mitigating the on-target anemia observed with CD47 blockade is critical to successful clinical development of CD47 targeting agents.

## Clinical Experience of CD47 Targeting Agents in AML and MDS

To date, three CD47 targeting agents have been evaluated clinically in AML and MDS. These include magrolimab (previously known as 5F9) (Forty Seven, Inc.), TTI-621 (Trillium Therapeutics), and CC-90002 (Celgene) (Table 2). Magrolimab and CC-90002 are IgG4 anti-CD47 antibodies while TTI-621 is an IgG1 SIRPα-Fc fusion protein. While clinical data have been reported for magrolimab in AML and MDS patients, no AML/MDS data have been reported for TTI-621 or CC-90002. A Phase 1a/b trial of TTI-621 in hematologic malignancies was initiated in 2016 that includes evaluation of multiple cancer types including AML and MDS patients. While data has been reported from this Phase 1 for other malignancies (30), no AML or MDS data has been reported to date. However, data has been reported for CC-90002 in AML/MDS (33). 24 patients with R/R AML and 4 patients with R/R MDS were treated (33). Four patients experienced a dose limiting toxicity (Grade 4 disseminated intravascular coagulation and Grade 4 cerebral hemorrhage, Grade 3 purpura, Grade 4 congestive heart failure and Grade 4 respiratory failure, and Grade 4 sepsis). 25% of patients discontinuing therapy due to an adverse event. The best overall response was stable disease in 2 MDS patients with no objective responses observed in either MDS or AML. Development of anti-drug antibodies occurred in 36% of patients by day 22. In October 2018, Celgene discontinued a Phase 1 trial evaluating CC-90002 in relapsed/refractory (R/R) AML and high risk MDS reporting that preliminary data did not offer a sufficiently encouraging profile for further dose escalation/expansion (NCT02641002). Given the availability of data, the following section outlines clinical data reported for magrolimab in AML and MDS patients.

**Table 2:** CD47/SIRPα targeting agents investigated in AML and/or MDS.

**Molecule**	**Magrolimab (5F9)**	**TTI-621**	**CC-90002**
**Sponsor**	**Forty Seven, Inc**.	**Trillium therapeutics**	**Celgene**
Mechanism of action	Anti-CD47 antibody, IgG4 isotype	SIRPα-Fc fusion protein. IgG1 isotype	Anti-CD47 antibody, IgG4 isotype
Studies in AML/MDS	Phase 1 trial of Anti-CD47 Antibody in Haematologic Malignancies (NCT02678338) Phase 1b trial of Hu5F9-G4 Monotherapy or Hu5F9-G4 in Combination with Azacitidine in Patients with Hematological Malignancies (NCT03248479)	Phase 1 trial of TTI-621 for Patients with Hematologic Malignancies and Selected Solid Tumors (NCT02663518)	Phase 1 trial of CC-90002 in Subjects with Acute Myeloid Leukemia and High-Risk Myelodysplastic Syndrome (NCT02641002)
Study results	Phase 1 results reported (31) Initial Phase 1b results reported (32)	AML and MDS patients were enrolled, no AML/MDS specific data have not been reported	Phase 1 results reported (33)
Study status	Phase 1 trial completed Phase 1b trial ongoing	Ongoing	Terminated
Developmental stage	Phase 2/registrational in MDS Phase 1b in AML	Phase 1	Discontinued in AML/MDS

Magrolimab was first evaluated in a first-in-disease multi-center Phase 1 trial as monotherapy in R/R AML conducted in the United Kingdom. Clinical data was reported as of June 2018 (31). 18 patients were enrolled in this dose escalation study. Five dose cohorts were explored from a magrolimab dose of 0.1–30 mg/kg twice weekly. The median age was 72 with a median of 2 prior therapies (range 1–5) with most patients enrolled being intermediate or poor cytogenetic risk. In terms of safety, no maximum tolerated dose or dose limiting toxicity was observed in these patients with dosing up to 30 mg/kg. The on-target anemia was the most common adverse event (AE) observed in 89% of patients experiencing a treatment-related anemia. The average hemoglobin drop was 0.95 g/dL with magrolimab dosing across all patients and timepoints. No patient discontinued therapy due to an AE and no drug-related deaths were reported. In terms of efficacy, 13 patients were evaluable for response with no objective responses observed. Fifty-Eight percent of patients had blast count reduction and 2 patients had biologic activity as evidenced by significant reduction in bone marrow cellularity and blast count (as seen in pre-clinical models) with 2 patients remaining in long term stable disease for over 10 months on therapy. Interestingly, one of these patients who was on therapy long-term with >50% blast count reduction had a significant increase in CD3 positive T cell infiltrates in the bone marrow, suggesting a potential adaptive immune response with magrolimab treatment. This clinical result is supported by the pre-clinical finding that anti-CD47 antibody can induce a CD8 T-cell specific anti-tumor response through cross-presentation of tumor antigens by macrophages to T cells upon tumor engulfment (11). Overall, magrolimab was well-tolerated in R/R AML with evidence of monotherapy activity, but insufficient for further development as a single agent.

Magrolimab was then investigated in a Phase 1b study both as a monotherapy safety run-in in R/R AML/MDS in the United States and in combination with azacitidine in untreated AML patients ineligible for induction chemotherapy and untreated MDS patients who are intermediate to very high risk by Revised International Prognostic Scoring System (IPSS-R) (32) (NCT03248479). Initial data from this study was reported in June 2019 (34) with most recent data reported in December 2019 (32). The trial reported two arms: (1) a magrolimab monotherapy safety run-in cohort in R/R AML/MDS; and (2) a magrolimab + azacitidine combination cohort in untreated AML ineligible for induction chemotherapy and untreated MDS intermediate to very high risk by IPSS-R. In the safety run-in monotherapy cohort 10 patients (6 AML and 4 MDS) were enrolled. Median age was 78 and 75 in AML and MDS patients, respectively, with a median of 3 prior therapies (for AML and 2 prior therapies for MDS. The safety of magrolimab monotherapy was similar to the Phase 1 experience with 1/10 (10%) of patients achieving an objective response. In the magrolimab + azacitidine combination cohort, 62 AML and MDS patients were treated. Median age in this untreated cohort was 74 and 70 years for AML and MDS patients, respectively. The majority of patients were poor cytogenetic risk and 70% of the AML patients had underlying myelodysplastic related changes. Thirty-one percent of MDS patients had therapy-related disease. Overall, the safety of magrolimab + azacitidine was well-tolerated with no MTD observed with up to 30 mg/kg weekly magrolimab dosing. The AE profile was similar to azacitidine monotherapy with no significant worsening of cytopenias. Regarding the on-target anemia, the average hemoglobin drop on magrolimab + AZA therapy was 0.4 g/dL and many patients improved their hemoglobin counts and had decreased transfusion requirements on therapy. Overall, 73% of AML patients and 44% of MDS patients who were transfusion-dependent at baseline became red blood cell transfusion independent on therapy. Treatment-related neutropenic fever occurred in <5% of patients with no treatment-related infections observed. Only 1 of 72 (1%) total patients treated with magrolimab (as monotherapy or in combination) discontinued due to an AE. No deaths were observed within the first 60 days of magrolimab + AZA treatment. With regards to efficacy, 46 AML/MDS patients were efficacy evaluable (Table 3). In AML patients, 14/22 (64%) achieved an objective response with 55% achieving a CR/CRi. In MDS 22/24 (92%) patients responded with 50% achieving a CR. The median time to response was rapid at 1.9 months. Additionally, deeper responses were observed as 60% of AML and 26% of MDS patients with abnormal cytogenetics at baseline achieved a complete cytogenetic response. Minimal residual disease (MRD) negativity was observed in 57% and 23% in AML and MDS responders, respectively. MRD was assessed by flow cytometry using a difference from normal technique with a lower limit of detection of 0.02%. Fifteen percent of evaluable patients treated on the combination proceeded to a successful allogeneic hematopoietic cell transplant. With a median follow-up of approximately 6.4 and 8.8 months for AML and MDS, respectively, no median duration of response was reached for either AML or MDS. The longest responses were ongoing over 14 months in several patients at time of presentation. Additional patient follow up will be needed to further determine whether these responses will be durable.

**Table 3:** Efficacy of magrolimab + azacitidine in untreated AML and MDS patients.

**Best overall response**	**1L AML (*N* = 22)**	**1L MDS (*N* = 24)**
ORR	14 (64%)	22 (92%)
CR	9 (41%)	15 (50%)
CRi	3 (14%)	–
PR	1 (5%)	0
MLFS/marrow CR	1 (5%)	8 (33%) [4 with marrow CR + HI]
HI	–	2 (8%)
SD	7 (32%)	2 (8%)
PD	1 (5%)	0

*ORR, objective response rate; CR, complete remission; CRi, complete remission with incomplete count recovery; PR, partial response; MLFS, morphologic leukemia free state; HI, hematologic improvement; SD, stable disease; PD, progressive disease. Responses were assessed according to 2017 European LeukemiaNet criteria for AML and 2006 International Working Group criteria for MDS. Data referenced was presented at the ASH annual meeting in 2019 (32)*.

Some initial correlative analyses were presented. First, given that CD47 is an LSC marker, putative CD34+CD38– LSC frequency in the bone marrow was assessed. LSC frequency was significantly reduced or eliminated in the majority of responding patients. Thus, magrolimab + azacitidine as a potential LSC targeting combination may lead to durable remissions, although longer clinical follow-up is needed. Second, magrolimab + AZA treatment led to a significant increase in CD4 and CD8 T cell infiltrates in the bone marrow of AML patients, suggesting that this combination activates an adaptive T cell anti-leukemic response. Lastly, magrolimab + AZA efficacy was reported for AML patients with a TP53 mutation, a particularly poor prognosis population. The CR/CRi rate in 9 TP53 mutant AML patients was 78% with 44% achieving CR and 33% achieving CRi. In addition, complete cytogenetic responses were observed in 67% of responders. Minimal residual negativity was observed in 57% of responders. Median duration or median survival was not reached with a median follow-up of 6.9 months. TP53 mutant variant allele frequencies were dramatically reduced or eliminated in all patients. While patient numbers and follow-up are limited, these data are encouraging as current available therapies are not effective in TP53 mutant AML. For example, for venetoclax + azacitidine, a current new standard of care in induction chemotherapy ineligible AML, the response rate was 47% with a median duration and overall survival of only 5.6 months and 7.2 months, respectively (35). Overall, magrolimab + AZA was well-tolerated, and while the number of patients and follow-up time were limited, the initial data are encouraging. Based on these data, magrolimab + AZA is being evaluated in a potential registrational multi-center study in untreated higher risk MDS patients.

## Novel Combinations With CD47 Targeting Agents and Future Directions in AML and MDS

While the clinical experience of CD47 targeting agents in AML and MDS is early, initial data with magrolimab + AZA demonstrates clinical proof-of-concept in these disease settings. While these data are encouraging, they raise a number of additional questions. First, which patients may preferentially respond to magrolimab + AZA? This question can be divided into three types of investigations: (1) biomarkers of response relating to the CD47/SIRPα pathway; (2) immunologic biomarkers; and (3) correlation of response by mutational profile. With regards to immunologic biomarkers, given that magrolimab activates several immune cell effectors (macrophages, dendritic cells, neutrophils, and T cells), the correlation of response to frequency and activation state of immune cell effectors in the bone marrow tumor microenvironment is important to characterize. Correlation of response with CD47/SIRPα expression, mutational profiling, frequency of macrophage and T cell effectors in the bone marrow microenvironment, and activation state of T cells in the bone marrow microenvironment are key to assess, and such studies are ongoing.

Second, what novel combinations will maximize efficacy with CD47 targeting agents? Given the broad mechanism of action of CD47 targeting agents as macrophage checkpoint inhibitors and potential leukemia stem cell targeting, this raises the question of whether additional combinations incorporating anti-CD47 agents will be effective. While multiple combination modalities can be pursued, it is worth highlighting three potential approaches. Such combination approaches will be emphasized with magrolimab given available clinical data, but may apply to other CD47 targeting agents. First, for magrolimab, significant enhancement of activity when adding azacitidine likely occurs due to the upregulation of pro-phagocytic signals on leukemia cells by azacitidine (23). This pro-phagocytic upregulation in large part occurs through direct cytotoxicity, but may also occur through hypomethylation or other mechanisms. This combination mechanism is particularly interesting in that other cytotoxic agents may also induce pro-phagocytic signals in a similar manner and would likely synergize with magrolimab. Thus, combinations of magrolimab with chemotherapy, particularly induction chemotherapy with 7 + 3 (7 days of cytarabine + 3 days of anthracycline) for newly diagnosed fit AML patients, or low dose cytarabine in AML patients unfit for induction chemotherapy, may lead to significant clinical responses. It should be noted that cytotoxic agents may also non-specifically upregulate pro-phagocytic signals on normal cells in addition to leukemic cells, thus leading to potential enhanced toxicities with CD47 therapeutic combinations. However, in an initial clinical experience with magrolimab + azacitidine, there was no significance enhancement of azacitidine toxicities, suggesting that the clearance of normal cells was not observed. Further investigation is needed to determine whether this selectivity extends to other cytotoxic agents. In addition, other agents that induce cell death, such as venetoclax, may also induce pro-phagocytic signals and thus synergize with magrolimab. Second, magrolimab can also synergize with tumor-targeting antibodies, given that such antibodies can provide an extrinsic pro-phagocytic signal through Fc receptor/macrophage-mediated antibody dependent cellular phagocytosis (5). This combination rationale has been clinically validated with magrolimab + rituximab combination therapy in non-Hodgkin's lymphoma (28). For AML or MDS, combination of magrolimab with tumor-targeting antibodies against leukemic antigens such as CD33 or CD123 may be efficacious. Third, magrolimab combination with T cell checkpoint inhibitors may lead to enhanced responses (11). Upon macrophage or dendritic cell-mediated phagocytosis of cancer cells by CD47 blockade, these phagocytes may present tumor antigens to T cells to induce anti-cancer T cell responses (11, 36). Therefore, combinations with T-cell checkpoint inhibitors (with anti-PD-1 or PD-L1 agents) may further augment T cell responses and enhance efficacy. Several pre-clinical studies have demonstrated that CD47 blockade in combination with T cell checkpoint inhibitors can enhance anti-tumor activity (37–39). A clinical trial evaluating magrolimab in combination with atezolizumab (an anti-PD-L1 antibody) has been initiated in R/R AML (NCT03922477).

## Conclusions

CD47 is a novel macrophage immune checkpoint that plays a broad role in cancer immune evasion across multiple cancer types and particularly in myeloid malignancies. In addition, CD47 has been identified as an LSC marker in AML. Therapeutic blockade of the CD47-SIRPα pathway has led to robust pre-clinical efficacy *in vivo* with several therapeutics in clinical development. While the clinical data of such agents in myeloid malignancies has been limited, initial data with magrolimab, a first-in-class anti-CD47 antibody, has been shown to be well-tolerated with encouraging efficacy results when combined with azacitidine in AML and MDS patients. These data highlight the clinical importance of targeting immune checkpoints such as CD47 and the critical role for macrophages in the pathophysiology of leukemic disease. As the scientific and clinical understanding of CD47 targeting agents matures, key questions are likely to be addressed including biomarker strategies for patient selection and the identification of effective therapeutic combinations with CD47 blockade. The next several years should prove fruitful in increasing the scientific and clinical understanding of CD47 targeting in myeloid malignancies.

## Disclosure

MC, J-PV, CT, and DF were employees of Forty Seven, Inc. KM and PG were employees of Forty Seven, Inc. at the time this work was conducted. IW and RM were co-founders, consultants, and serve on the Board of Directors of Forty Seven, Inc. The authors declare that this study received funding from Forty Seven, Inc. and were conducted with employees of Forty Seven, Inc.

## Author Contributions

MC, CT, J-PV, IW, and RM wrote the manuscript. DF, KM, PG, and JL performed experiments included in the manuscript.

### Conflict of Interest

This study received funding from Forty Seven and was conducted with employees of Forty Seven, Inc., MC, CT, DF, KM, PG, JL, and J-PV. Since employees or affiliates of Forty Seven conducted experiments and wrote the manuscript, the funder was involved in the study design, collection, analysis, interpretation of data, and writing the article. The remaining authors declare that the research was conducted in the absence of any commercial or financial relationships that could be construed as a potential conflict of interest.
